# Adverse events associated with Covishield vaccination among healthcare workers in a tertiary hospital in South India

**DOI:** 10.1016/j.jvacx.2022.100210

**Published:** 2022-08-28

**Authors:** Dipu T Sathyapalan, Merlin Moni, Preetha Prasanna, Vishal Marwaha, Sai Bala Madathil, Fabia Edathadathil, Sony A. Jose, Sheela Pavithran, Rajasree Muralikrishanan, Nigith Ramachandran, Roshni P R, Tinu T S, Anjana S. Nair, Sanitha Kuriachan, Princy Louis Palatty

**Affiliations:** a Division of Infectious Diseases, Department of General Medicine, Amrita Institute of Medical Sciences, Amrita Vishwa Vidyapeetham, Kochi, Kerala, India; b Amrita Institute of Medical Sciences, Amrita Vishwa Vidyapeetham, Kochi, Kerala, India; c Department of Rheumatology, Amrita Institute of Medical Sciences, Amrita Vishwa Vidyapeetham, Kochi, Kerala, India; d Department of Infection Control and Epidemiology, Amrita Institute of Medical Sciences, Amrita Vishwa Vidyapeetham, Kochi, Kerala, India; e Department of Nursing, Amrita College of Nursing, Amrita Institute of Medical Sciences, Amrita Vishwa Vidyapeetham, Kochi, Kerala, India; f Amrita College of Nursing, Amrita Institute of Medical Sciences, Amrita Vishwa Vidyapeetham, Kochi, Kerala, India; g Department of Community Medicine, Amrita Institute of Medical Sciences, Amrita Vishwa Vidyapeetham, Kochi, Kerala, India; h Department of Pharmacy Practice, Amrita School of Pharmacy, Amrita Institute of Medical Sciences, Amrita Vishwa Vidyapeetham, Kochi, Kerala, India; i ADR Monitoring Centre, Amrita Institute of Medical Sciences, Amrita Vishwa Vidyapeetham, Kochi, Kerala, India; j Department of Pharmacology, Amrita Institute of Medical Sciences, Amrita Vishwa Vidyapeetham, Kochi, Kerala, India

## Abstract

**Background:**

Vaccination is the most important prophylactic measure taken to curb COVID-19 pandemics. This study was undertaken to throw light on the safety of Covishield vaccine among health care workers (HCWs) and to assess the co-variates associated with incidence of adverse events.

**Methods:**

This prospective observational study was conducted in a tertiary care center in South India as part of the HCW vaccination drive. All consenting HCWs who received the first dose of Covishield vaccine and developed ADRs were included in this study. After vaccination, all beneficiaries were monitored for AEFI for a period of half an hour and later followed up through telephone and google survey forms on day 2 and day 7 of vaccination. The data was subsequently collated into spreadsheet format and analyzed.

**Results:**

The study included 1264 consenting healthcare workers who were predominantly youth, aged 15–24 years (n = 583, 46 %) and with a female preponderance of 76 % (n = 960). Past history of COVID-19 infections was reported among 4.6 % (58) of the study population. Postvaccination symptoms were majorly reported during the first (40 %) and second day (44 %) after vaccination with a high prevalence of both local (n = 1083, 85 %) and systemic symptoms (n = 1065, 84 %). The mean duration of symptoms was observed to be 1.4 ± 0.81 days post vaccination. Symptoms were observed significantly high among females (76.7 %, p = 0.013). The prevalence of systemic (88 % vs 80 %) (p < 0.001) and allergic symptoms (7 % vs 3 %; p = 0.03) were observed to be significantly high among respondents with <25 years of age. The systemic and allergic symptoms following vaccination were reported to be low among healthcare workers who had a previous history of COVID-19 infection.

**Conclusion:**

COVID vaccination has been observed to be safe and well tolerated with more systemic symptoms reported among younger age group and females.

## Introduction

COVID-19 pandemic has escalated to devastating proportions with 50 crore confirmed cases globally as of April 2022 based on WHO dashboards and death toll hitting 62 lakhs worldwide [1]. All governmental organizations have adopted preventive strategies comprising of lock down measures, social distancing, appropriate sanitization, and face masking to curb the spread of the virus. The advent of vaccine development and its subsequent administration among the population have been believed to be the major and effective prophylactic measure amidst the escalating subsequent waves of COVID-19 infections.

**Fig. 1:**
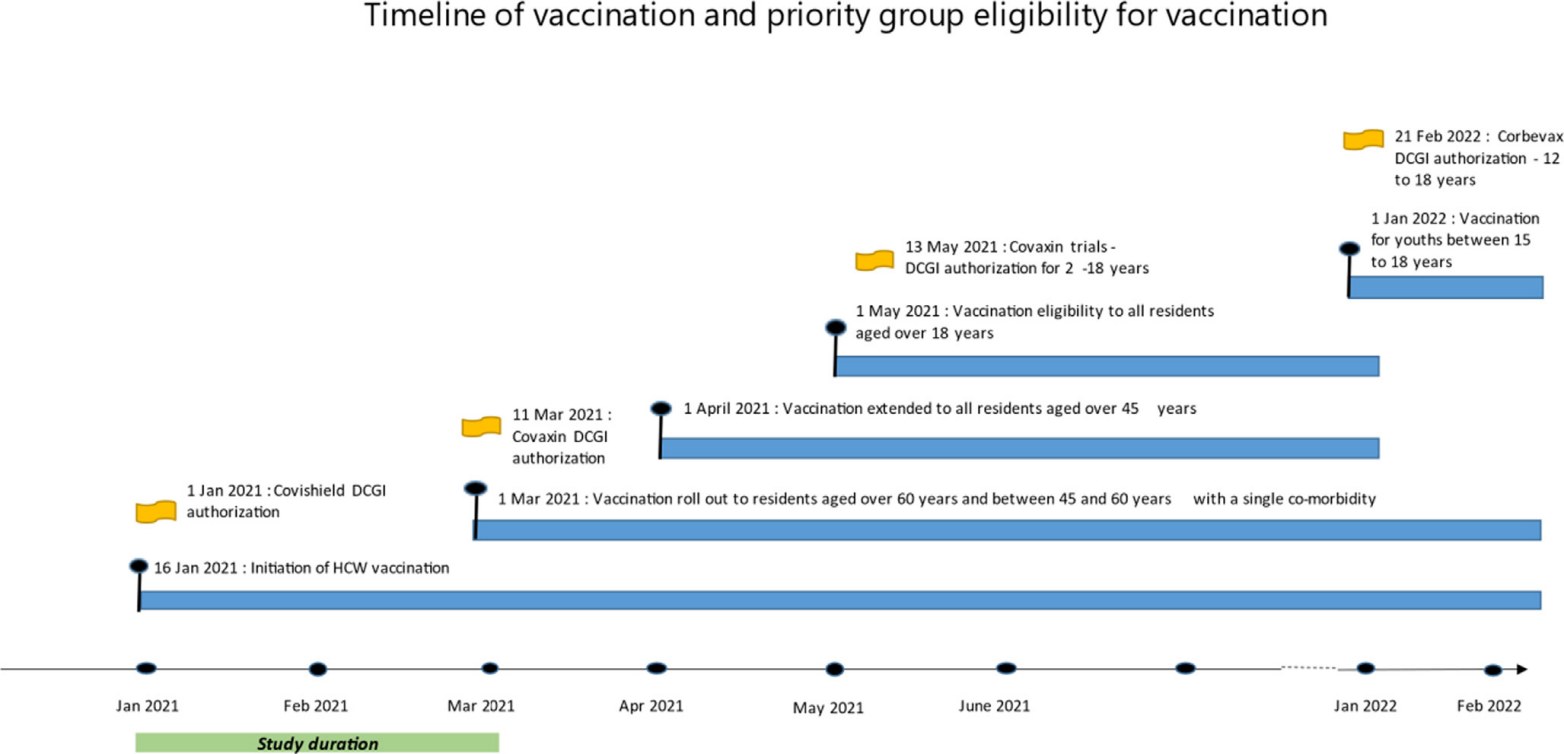
xxxx.

India is touted to be one of the countries with the largest vaccine roll-out targets presently administering vaccines according to the recommendations of National Expert Group on Vaccine Administration for COVID-19 (NEGVAC) [2], [3], [4], [5]. Initially Covishield and Covaxin manufactured by Serum Institute of India and Bharat Biotech Ltd respectively were administered in the country. India has administered 187 crores of total COVID-19 vaccination doses with first and second doses vaccination coverage to almost 100 and 85 crores respectively till April 2022 [4], [6]. The timeline for vaccination for priority population groups have been depicted in Fig. 1.

The apprehension over the safety and efficacy of the COVID-19 vaccine looms high in the mind of the public [7]. Being on new vaccines, post vaccination safety surveillance is essential to quantify the adverse events associated with COVID-19 vaccination for assessing causality. The COVID-19 vaccines underwent clinical trials for relatively shorter periods and in small pockets of population to fulfil the accepted criteria for evaluation and safety assessment. When the vaccines are massively rolled out to the wider population, vaccines are encountering a vast heterogeneous pharmacogenetic pool. The adverse events that would follow such vaccination efforts could be variable. Hence, stringent monitoring of every minor as well as serious adverse events need to be reported and analyzed. Alternatively, Adverse Effects Following Immunization (AEFI) may affect healthy individuals and should be promptly identified to allow additional research and appropriate action to take place. Timely detection and reporting of adverse events following COVID-19 vaccination is the first step in ensuring the continued safety of the vaccine, immunization safety surveillance and response. In the COVID-19 vaccination context, surveillance systems need to be prepared for identifying and responding to both AEFI and Adverse Event of Special Interest (AESIs) as well as other safety events that may cause public concern. AEFI is defined as an untoward medical occurrence that may or may not have a causal association with the vaccine administration. The role of vaccine safety surveillance during COVID-19 vaccine introduction is to facilitate the early detection, investigation, and analysis of AEFIs and AESIs of notable medical concern to ensure an appropriate and rapid response. This will decrease the negative impact of these events on the health of individuals and the immunization programs and maintain the confidence of health care professionals and the general population.

Covishield and Covaxin were the initially approved vaccines in the state of Kerala during vaccine roll out. This study aims to report the AEFI of Covishield vaccine and associated co-variates among healthcare workers (HCW) post vaccination.

## Methods

### Study design and setting

The prospective observational study was conducted in a 1300 bedded tertiary care academic center in South India as part of the vaccination drive for healthcare workers which commenced on Jan 19, 2021. The data collection was done for a period of 5 weeks. Ethical approval for the study was obtained from the Institutional ethics committee (letter No: ECASM-AIMS-2021-139).

### Study population

The vaccination drive had included approximately 8000 HCWs comprising staff, faculty and students of our academic tertiary care hospital attending our vaccination clinic. All consenting HCWs who received the first dose of vaccine and developed Adverse Drug Reactions (ADRs) were included in this study as per the study inclusion criteria, adopting a consecutive sampling technique.

### AEFI surveillance process

We had conducted a 30-minute observation for adverse events following vaccine administration for all HCWs as per the standard protocol at our vaccination clinic [5]. An onsite medical team was deployed at the clinic for detection and stabilization of immediate adverse events during the observation period. In addition, an education on AEFI was provided by a trained medical staff to the HCWs on exit process.

The vaccination center being part of a tertiary care hospital, a physician was available at the center for providing clinical care to vaccine recipients who developed AEFI related symptoms at designated patient observation rooms identified for the purpose of managing immediate ADRs.

The rapid response preparedness included availability of critical care physicians on site with emergency medicine consultations, designated patient observation rooms and emergency medical equipment such as crash carts. A medical helpline was available for 24-hours for the vaccine beneficiaries to seek clinical care for any untoward adverse events. The aforementioned activities were applicable to all HCWs undergoing vaccination.

### AEFI reporting

Reported adverse events (AEs) were recorded by trained pharmacists of institutional vaccination team stationed at the vaccination clinic in standard WHO COVID-19 AEFI reporting forms. Those HCWS who developed immediate ADR were contacted via phone after 24 h and were reassessed. All the consenting vaccination beneficiaries were followed up for adverse events by disseminating indigenously prepared, validated AEFI google forms to their registered mobile numbers.

Data collection in AEFI forms were also done by structured telephonic interview for those respondents who are at technological disadvantage to use google forms. Telephonic interview was done by institutional vaccination safety surveillance team on day 2 and day 7 of vaccination [8]. The institutional vaccination safety surveillance team is a multidisciplinary group led by an Infectious Disease physician as COVID nodal officer in charge for vaccination drive and comprising of infection prevention control (IPC) representative, nurses and clinical pharmacists. All AEFIs were immediately reported by the team to Regional Training Centre, under Pharmacovigilance programme, India (PvPI). This study is carried out under the initiative of the Regional Training Centre (RTC) which involves pharmacologists, hospital administrators, clinical specialists, nurses and pharmacists, at Amrita Institute of Medical sciences, under pharmacovigilance program of India. The data was subsequently collated into spreadsheet format and analyzed.

### Pharmacovigilance programme of India

Pharmacovigilance programme of India (PvPI) started formally in 2010 to create a nation-wide system for drug safety reporting. Capturing adverse event reports is the primary objective of PvPI, which helps regulatory authorities to make informed decisions and communicate the safety information to different stakeholders. Currently there are around 250 functioning Adverse Drug Monitoring centres and 12 regional training centres in the country as part of the Pharmacovigilance Programme of India.

Although AEFI Surveillance System in India was active since its inception from 1986, it is added to the PvPI in 2015 to monitor vaccine related adverse events. When it comes to COVID-19 vaccines which did not undergo clinical trials of adequate duration and was given emergency use authorization, safety data on COVID vaccine needs scrutiny. Both minor and serious AEFIs is reported to PvPI, while serious AEFIs are also reported to district immunization officer to take remedial measures. PvPI further coordinates AEFI cases with national level committee AEFI for reporting and inquiry.

### Sample size estimation

Considering the lack of robust data from literature on AEFI as part of vaccination safety surveillance in Indian context, the sample size of the proposed study was estimated based on the result of pilot study conducted with 10 samples. Based on the proportion of adverse events following COVID-19 vaccination such as Local Symptoms (30 %), Systemic Symptoms (70 %) and Allergic Symptoms (0 %) in healthcare workers obtained from pilot study and with 10 % relative precision and 95 % confidence, the minimum sample size comes to 896, 165, 0 so the minimum required sample size required was 896. Since we didn’t want to miss out any rare ADRs, we included all the 1264 consenting HCWs who developed ADRs in our study.

### Statistical analysis

Continuous variables were expressed as mean ± standard deviation. Categorical and nominal variables were expressed as frequencies and percentages. To test the normality of data, Kolmogrov Smirnov or Sharpiro Wilcoxon test was applied. Chi square test is used to compare frequencies. Logistic regression analysis was performed to identify predictors of symptoms. Data analysis was done using SPSS v. 17 (IBM, US). p < 0.05 was considered to be significant.

## Results

An estimate of the total AEFI events pertaining to the 8000 HCWs vaccinated was not available with us, as all of them did not respond back. We estimate a response rate of approximately 45 %, considering the controlled roll out to be 2800. The post vaccination safety surveillance included 1264 HCWs from our institution who provided consent for study participation.

### Demographics

**Fig. 2:**
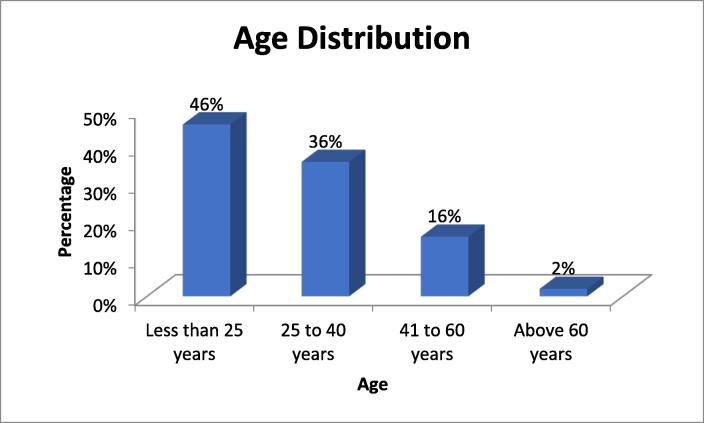
xxxx.

**Fig. 3:**
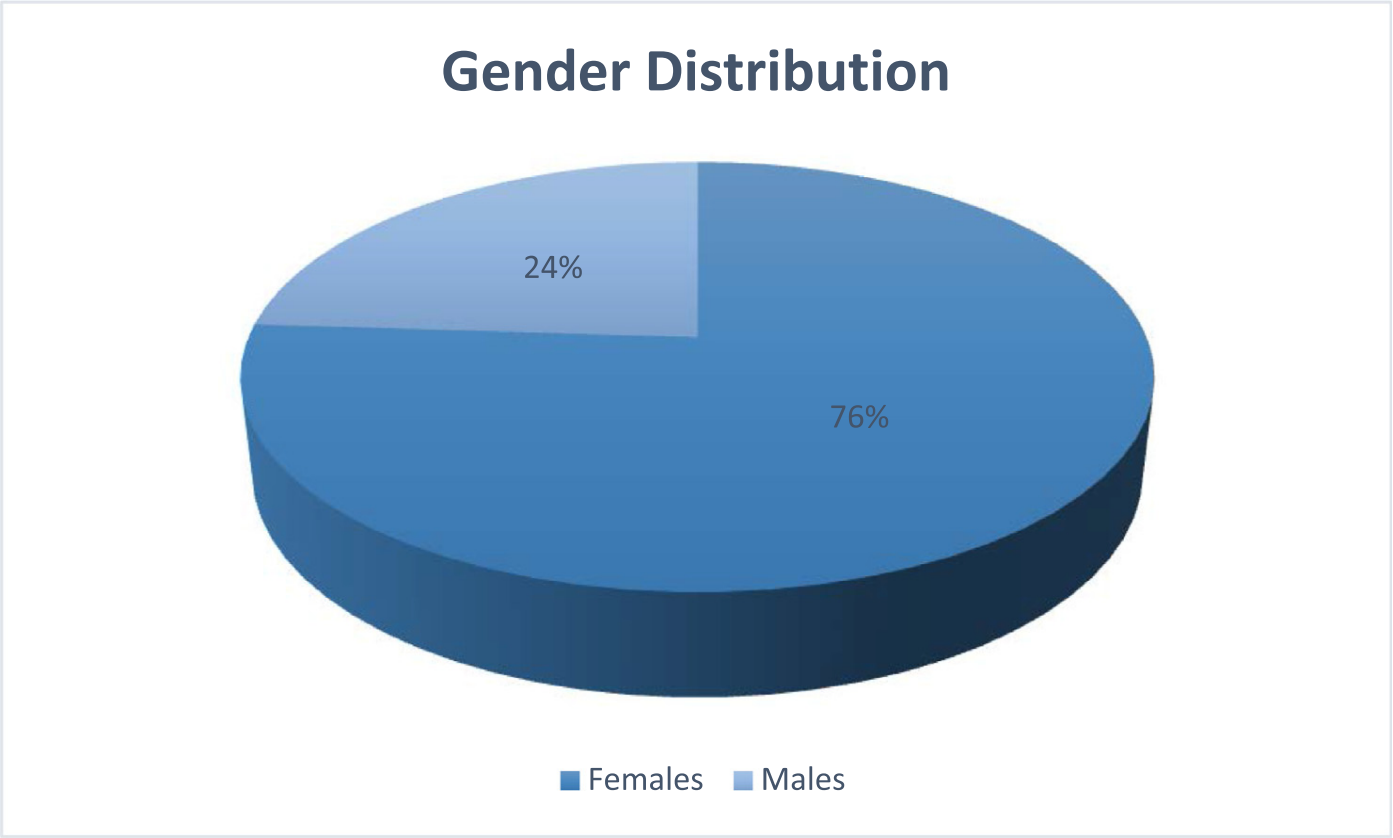
xxxx.

**Fig. 4:**
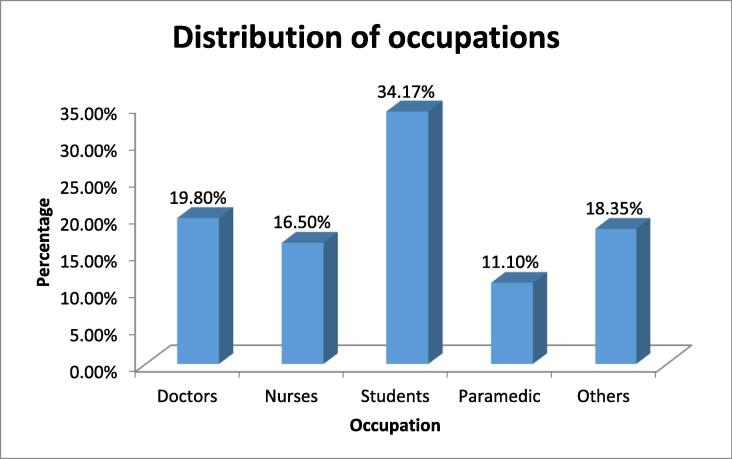
xxxx.

The baseline characteristics of the healthcare workers enrolled into the study is given in Table 1 and Fig. 2, Fig. 3. Our HCW study group comprised predominantly of youth aged 15–24 years (n = 583, 46 %) with a mean age of 29.4 ± 25.0 years. Adults aged <25 years account for 46 % (n = 583) of the study population (Fig. 2). 76 % (n = 960) were observed to be females (Fig. 3). Doctors (n = 251, 19.8 %), nurses (n = 209, 16.5 %) and students (n = 432, 34.17 %) majorly comprised the occupational profile of the study population (Fig. 4). Past history of COVID infection was reported by 4.6 % (n = 58) of the surveyed respondents. Voluntary intake of paracetamol was reported among 71.2 % (n = 900).

**Table 1:** Baseline characteristics.

Characteristics	Mean ± S D
Age in years (Mean ± SD)	29.4 ± 25.0
Mean duration of symptoms	1.4 ± 0.81 days
	**n (%)**
Pre-existing co-morbidities	134 (10.6 %)
Past allergic history	50 (4 %)
Regular medications	172 (13.6 %)
COVID positivity	58 (4.6 %)
Management	
Voluntary intake of paracetamol	900 (71.2 %)
Medication prescription	9 (0.7 %)
Hospitalization due to allergy	14 (1.1 %)

### Symptomatic profile post vaccination

**Fig. 5:**
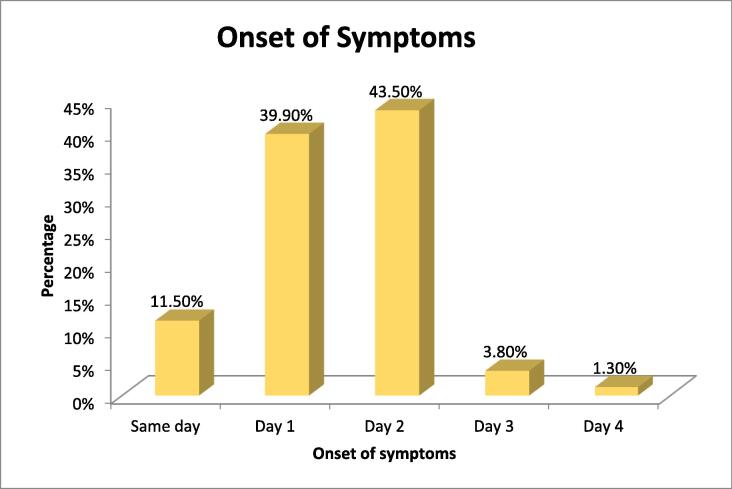
xxxx.

**Fig. 6:**
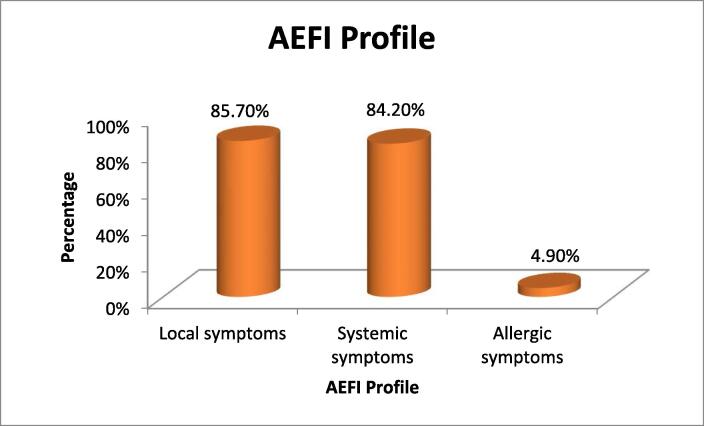
xxxx.

Symptoms occurring after vaccination were majorly reported during the first (40 %) and second day (44 %) of vaccination (Fig. 5). The mean duration of symptoms was observed to be 1.4 ± 0.81 days after vaccination (Table 1). Symptomatic profile revealed high prevalence of both local (n = 1083, 85 %, 95 % CI 83 %–87 %) and systemic symptoms (n = 1065, 84 %, 95 % CI 82 %–86 %) (Fig. 6). Local pain was reported by 78.6 % (9 9 3) of the study population. The most commonly reported systemic symptom post vaccination was fever (n = 692, 54.7 %) followed by headache (n = 629, 49.8 %), fatigue (n = 512, 40.5 %) and myalgia (n = 434, 34.3 %). Gastrointestinal (GI) symptoms were reported less frequently and included nausea (n = 142, 11.2 %), loose stools (n = 49, 3.9 %) and vomiting (n = 60, 4.7 %) (Table 2). Among the HCWs who reported systemic symptoms, 59 % (n = 751) reported at least 2 symptoms and 37 % (n = 471) had at least 3 or more symptoms following vaccination.

**Table 2:** AEFI detailed symptom profile.

Symptoms	n(%)
*Local symptoms*	1083(85.6 %)
Local pain	993(78.6)
Tenderness	304(24 %)
Swelling	44(3.5 %)
Others	52 (4.1 %)
*Allergic symptoms*	63 (4.9 %)
Rash	13(1 %)
Urticaria	2(0.2 %)
Itching	34(2.7 %)
Angioedema	1(0.1 %)
Anaphylaxis	1(0.1 %)
Others	16(1.3 %)
*Systemic symptoms*	1065 (84.2 %)
Fever	692(54.8 %)
Headache	629(49.7 %)
Myalgia	435(34.4 %)
Sorethroat	92(7.3 %)
Fatigue	512(40.5 %)
Nausea	142(11.2 %)
Vomiting	60(4.7 %)
Loose stools	49(3.9 %)
Shortness Of Breath	33(2.6 %)
Psychological stress	27(2.1 %)
Palpitation	14(1.1 %)
Others	57(4.5 %)

Fever and headache were the most prevalent symptomatic complex (n = 429, 33.9 %), followed by fever and fatigue (n = 339, 26.8 %). Allergic symptoms were reported by only 5 % (n = 63, 95 % CI) HCWs among the study population. Two patients developed immediate side effects i.e., within half an hour following vaccination. Among the serious adverse events, there was one case of angioedema and another case of anaphylaxis (Table 2).

**Fig. 7:**
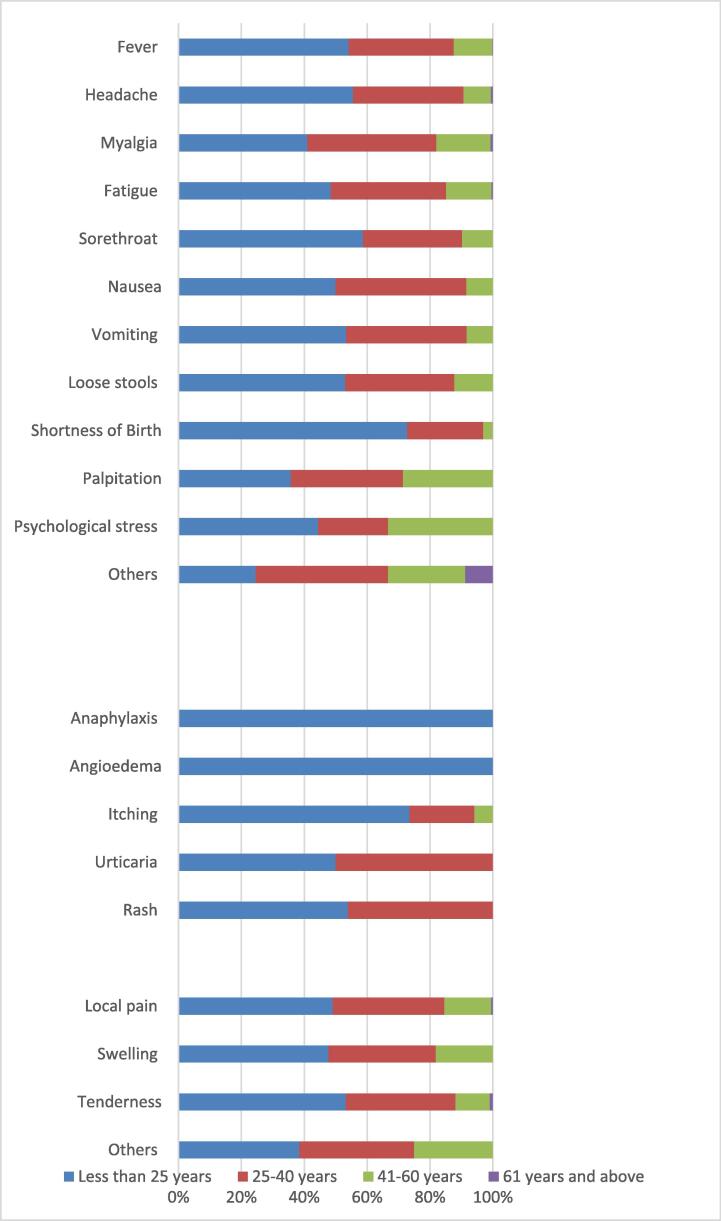
Age wise distribution of systemic, allergic and local symptoms.

The age-based distribution of local, systemic and allergic symptoms was depicted in Fig. 7. The prevalence of systemic (88 % vs 80 %) (p < 0.001) and allergic symptoms (7 % vs 3 %; p = 0.03) were observed to be significantly higher among respondents with <25 years of age.

**Fig. 8:**
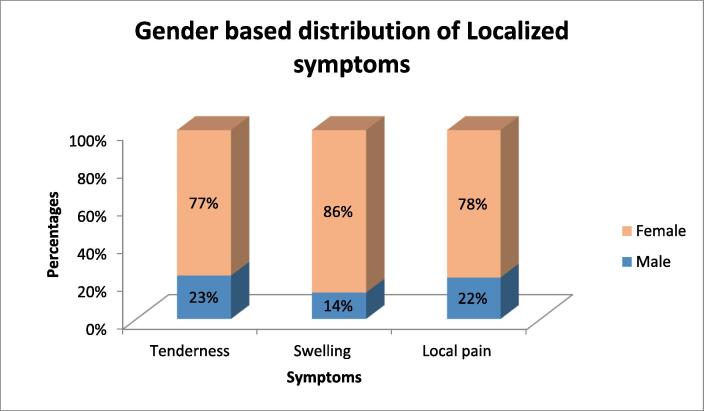
xxxx.

**Fig. 9:**
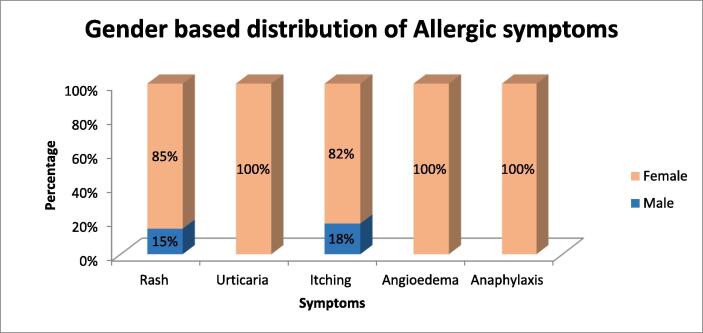
xxxx.

**Fig. 10:**
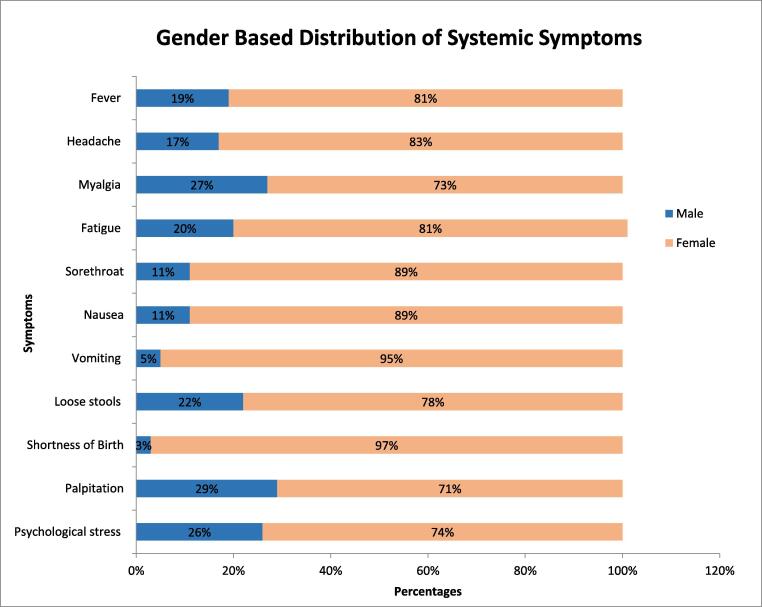
xxxx.

Similarly, a significantly higher incidence of symptoms post vaccination was reported among females (76.7 %) in comparison to males (69.7 %) (p = 0.013) (Table 3, Fig. 8, Fig. 9, Fig. 10). The symptoms such as fever (80.6 %), headache (83.5 %), sore throat (89 %), fatigue (80.3 %), nausea (89 %), vomiting (95 %) and shortness of breath (97 %) were reported high among females (p < 0.05). GI symptoms were significantly higher among females (87.2 %) relative to males (73.8 %); p < 0.001). No significant association was observed for psychological stress among the respondents and their gender (p = 0.48). Psychological stress was similar across all health professional categories, including Frontline Healthcare Workers (FHCWs).

**Table 3:** Gender based distribution of post vaccination symptoms.

Post vaccination symptoms	Males	Females	OR	p
Localized symptoms	n(%)	n(%)		
Tenderness	69 (23)	235 (77)	1.1 (0.8–1.49)	0.291
Swelling	6 (14)	38 (86)	2.0(0.85–4.8)	0.06
Local pain	218 (22)	775 (78)	1.65 (1.22–2.22)	0.001
Allergic profile				
Rash	2 (15)	11 (85)	1.75 (0.38–7.94)	0.36
Urticaria	0	2 (100)	–	0.5
Itching	6 (18)	28 (82)	1.49 (0.61–3.63)	0.25
Angioedema	0	1(100)	–	0.75
Anaphylaxis	0	1 (100)	–	0.75
Systemic symptoms				
Psychological stress	7 (26)	20 (74)	0.9 (0.3–2.1)	0.48
Palpitation	4 (29)	10 (71)	0.78 (0.2–2.5)	0.445
Shortness of Birth	1 (3)	32 (97)	10.4 (1.42–76.7)	0.001
Loose stools	11 922)	38 (78)	1.09 (0.55–2.17)	0.47
Vomiting	3 (5)	57 (95)	6.3 (1.9–20.3)	<0.001
Nausea	15 (11)	127(89)	2.9 (1.6–5.09)	<0.001
Sorethroat	10 (11)	82 (89)	2.7 (1.4–5.3)	0.001
Fatigue	101 (20)	411 (80)	1.5 (1.14–1.97)	0.002
Myalgia	115 (27)	319 (73)	0.81(0.62–1.06)	0.081
Headache	104 (17)	525 (83)	2.3 (1.02–3.0)	<0.001
Fever	134 (19)	558 (81)	1.76 (1.35–2.28)	<0.001

No significant association was observed between the incidence of allergy and pre-existing co-morbidities (p = 0.35). Surprisingly, past history of allergy showed no significant association with occurrence of hypersensitivity reactions among vaccine beneficiaries (p = 0.63). Among respondents with local and systemic symptoms, only 3.8 % had history of allergies. On the contrary, 5 % of respondents had history of allergies among those without local and systemic symptoms (p = 0.41). Our study did not observe any significant association between the incidence of allergic symptoms in patients with past history of allergy. The rate of anaphylactic reactions among recipients of mRNA based vaccine were reported to be very rare at 4.4 per million [9]. Vaccines based adenoviral vectors such as Covishield were reported to have even relatively lower anaphylactic rates (OR 0.47; 95 % CI 0.33–0) [10]. Our study observations also seem to support the data. International GRADE recommendations based on systematic meta-analysis considers anaphylaxis and allergic events to be rare on adjudicated data and self-reported allergies need not be regarded as a risk for vaccine administration [11].

Our study population revealed a significantly higher proportion of prior COVID-19 infections among respondents aged more than 25 years (7.2 % vs 1.5 %; p < 0.001). The systemic and allergic symptoms following vaccination were reported low among respondents who had a previous history of COVID-19 infection (Vaccination was done 3 months after being COVID 19 negative).

The GI symptoms were observed to be significantly low (6.9 % vs 16.5 %, p = 0.03) among patients who had a history of COVID-19 infection. The systemic symptoms among patients with previous history of COVID-19 infection was observed to be 81 % in comparison to 84 % observed among respondents without prior COVID-19 infections. The presence of local symptoms among respondents with previous history of COVID (3.7 % vs 9.9 %; p = 0.001) were observed to be significantly low. The respondents who had antihypertensive and anticoagulants as regular medications were observed to have significantly lower systemic symptoms at 65.6 % compared to those without medications (84.7 %, p = 0.007).

Logistic regression analysis performed to identify predictors of systemic symptoms identified respondents with age <25 years and female gender to be 1.79 (p = 0.003, 95 % CI 1.21–2.63) and 1.66 (p = 0.003, 95 % CI 1.19–2.31) times respectively more likely to develop systemic symptoms in comparison to respondents aged more than 25 years and male.

## Discussion

The knowledge in AEFI is primarily reported from clinical trials, but real-world data about the same has been scarce. This prospective study on AEFI was done by the institutional multidisciplinary team among 1264 healthcare workers following their first dose of Covishield vaccination. Assessment of AEFI at 48 h would provide insight into early AEFI and local symptoms. The standard AEFI surveillance protocol across the country specifies monitoring symptoms at 7 days post COVID vaccine administration. This would ensure comprehensive capture of AEFI and detailing of the clinical symptoms developed over the week in compliance to our study protocol enabling the evaluation of systemic symptoms. Additionally, identification of spectrum of AEFI in the given time points are important in devising rectification strategies specific to these time limes.

The prevalent symptomatic profile comprising of fever, fatigue, headache and myalgia observed among the respondents surveyed are similar to the most commonly observed systemic adverse reactions reported for the ChAdOx1 nCoV-19 vaccine developed by Oxford University [12], [13]. The reported reactogenicity rate of the vaccine varied from 60 to 88 % [13]. In our population, it was interesting to note that no significant association was observed in the incidence of allergic symptoms in patients with past history of allergy. It could possibly be due to low subset of allergic population which needs further exploration. We believe that this finding would be an aid to eradicate the hesitancy for vaccination in people with history of allergies. In addition, our study demonstrated the tolerability of the vaccine in patients taking medications which included antihypertensives and anticoagulants.

The study was conducted during the month of January–February 2021, at the post pandemic time point where COVID vaccinations were commenced among priority groups identified by the government of India. Health workers were one of the priority groups among whom the vaccine roll-out commenced. The demographics of our study population comprising of healthcare workers represented a very low proportion of HCW aged >60 years (2 %). A high occurrence of AEFI related symptoms with a higher prevalence of systemic symptoms among beneficiaries aged <25 years and of female gender were observed in our study. Our observations pertaining to higher prevalence of systemic symptoms among younger population and female gender are in lieu of the published reactogenicity reports [14], [12], [15]. This suggests vaccine reactogenicity declines with age [12], [16], [17], [18]. Vaccine reactogenicity is elicited as part of the heightened immune response generated in the recipients following vaccination. The ageing of the immune system termed as immune-senescence results in poor immunogenic response among older population in response to vaccination as revealed by low surrogate biomarkers of immunogenicity. Inflammatory responses post vaccination would also be consequently reduced in older population due to the declined immunogenic potential. Our study observations are in lieu of reports stating lower reactogenicity rates implied by lower AEFI rates among older people compared to younger population. We could not find any possible explanation for higher reactogenicity among females. However, our study demonstrated lower AEFI related symptoms among respondents with past history of COVID-19 infections, contrary to previous studies that reported increased risk of side effects with prior COVID-19 infections attributable to higher antibody titres [15], [19], [20], [21]. This is also interesting in the view of a significantly low proportion of prior COVID-19 infections among respondents aged <25 years who reported significantly high prevalence of AEFI related symptoms. AEFI related symptoms were reported to be lower among mRNA platform-based vaccine recipients receiving second dose vaccination with prior COVID history in comparison to those without a past history of COVID [12], [19], [22]. Though systemic responses are documented to be higher among vaccine recipients with past history of COVID, our study did not observe significant association between the incidence of AEFI and past history of COVID, this could be explained by the relatively higher rates of AEFI observed in our cohort (81 %) as compared to the reported AEFI rates in other studies (53.1 %).

The national policy had prioritized healthcare workers as the first recipients of vaccines in the current vaccination drive [23]. However considerable resistance was met in the initial stages due to fear of adverse events. As the fear of adverse events was the major contributory factor towards vaccine hesitancy, formal tracking for adverse events and reporting are of paramount importance [24]. AEFI monitoring could lead to better acceptance and psychological support as telephonic assessments reassured patients with relevant medical information and counseling. However, establishment of a system for tracking is challenging whilst in established tracking systems, the reporting is primarily active reporting, which is a main barrier in capturing AEFI in Low-And Middle-Income Countries (LMIC) settings [25]. AEFI reporting systems often capture the SAEs which constitutes a small minority of events and the events occurring in majority of the recipients are often overseen.

## Conclusion

Our study has demonstrated that AEFI were very common and mostly mild with limited serious side effects. COVID vaccination has been observed to be safe and well tolerated with more systemic symptoms reported among younger age group and females.

We believe the introduction and re-enforcement of active surveillance across healthcare systems undertaking vaccination campaigns would enable robust monitoring and reporting of non-SAEs. The system needs to be actively engaged in consistent follow-up of vaccine recipients and involve with hospital pharmacovigilance division for reporting. Promoting an active reporting of AEFI by vaccine recipients and enhancing seamless interaction and addressal of the AEFI by healthcare providers through interactive online platforms could maximize AEFI reporting. The re-enforcement of active surveillance systems in conjunction with pharmacovigilance teams and digitization would minimize underreporting on non-SAEs.

## Limitations

This survey was done in English language, which might negatively affect the response from those with low language proficiency.

## Declaration of Competing Interest

The authors declare that they have no known competing financial interests or personal relationships that could have appeared to influence the work reported in this paper.
